# Perception of social media behaviour among medical students, residents and medical specialists

**DOI:** 10.1007/s40037-021-00660-1

**Published:** 2021-04-07

**Authors:** Sebastiaan A. Pronk, Simone L. Gorter, Scheltus J. van Luijk, Pieter C. Barnhoorn, Beer Binkhorst, Walther N. K. A. van Mook

**Affiliations:** 1 Faculty of Health, Medicine & Life Sciences, Maastricht University, Maastricht, The Netherlands; 2 Academy for Postgraduate Medical Training, Maastricht University Medical Centre, Maastricht, The Netherlands; 3 Department of Internal Medicine, Division of Rheumatology, Maastricht University Medical Centre, Maastricht, The Netherlands; 4 Department of Public Health and Primary Care, Leiden University Medical Centre, Leiden, The Netherlands; 5 Department of Intensive Care Medicine, Maastricht University Medical Centre, Maastricht, The Netherlands; 6 School of Health Professions Education, Maastricht University, Maastricht, The Netherlands

**Keywords:** Social media, Online professionalism, Medical students, Residents, Medical specialists

## Abstract

**Introduction:**

Behaviour is visible in real-life events, but also on social media. While some national medical organizations have published social media guidelines, the number of studies on professional social media use in medical education is limited. This study aims to explore social media use among medical students, residents and medical specialists.

**Methods:**

An anonymous, online survey was sent to 3844 medical students at two Dutch medical schools, 828 residents and 426 medical specialists. Quantitative, descriptive data analysis regarding demographic data, yes/no questions and Likert scale questions were performed using SPSS. Qualitative data analysis was performed iteratively, independently by two researchers applying the principles of constant comparison, open and axial coding until consensus was reached.

**Results:**

Overall response rate was 24.8%. Facebook was most popular among medical students and residents; LinkedIn was most popular among medical specialists. Personal pictures and/or information about themselves on social media that were perceived as unprofessional were reported by 31.3% of students, 19.7% of residents and 4.1% of medical specialists. Information and pictures related to alcohol abuse, partying, clinical work or of a sexually suggestive character were considered inappropriate. Addressing colleagues about their unprofessional posts was perceived to be mainly dependent on the nature and hierarchy of the interprofessional relation.

**Discussion:**

There is a widespread perception that the presence of unprofessional information on social media among the participants and their colleagues is a common occurrence. Medical educators should create awareness of the risks of unprofessional (online) behaviour among healthcare professionals, as well as the necessity and ways of addressing colleagues in case of such lapses.

**Supplementary Information:**

The online version of this article (10.1007/s40037-021-00660-1) contains supplementary material, which is available to authorized users.

## Introduction

Ideally, a doctor possesses a variety of competencies, including medical knowledge, technical skills and being responsible and respectful towards patients. Governing bodies and patients expect doctors to behave in a professional manner, respecting the values and norms of the profession as defined by most professional medical organizations [1–3]. Nevertheless, a substantial part of patient complaints filed against healthcare professionals are related to poor communication and unprofessional behaviour [4]. Disciplinary measures against doctors have been shown to be associated with unprofessional behaviour during their undergraduate education [5–8]. Consequently, there is an increasing interest in teaching and assessment of professional behaviour in medical education [9–11].

With the exponential rise of the popularity and use of web-based technology over the last decade, observable behaviour is no longer limited to real-life events, but also visible on social media. Social media are defined as a group of internet-based applications that allow the user to create and exchange user-generated content within a digital network or community [12]. Social media are increasingly used in healthcare and medical education, which affects students’ interaction with peers, teachers, healthcare professionals and patients [11, 13–16]. For example, social media by which patients receive correct information about their condition, thus stimulating a more equal communication between patients and their healthcare professionals, can be used to improve self-management of patients [13].

In a response to increasing social media use, medical governing bodies in the United States [17], the United Kingdom [18], and the Netherlands have published guidelines on its use [19]. In the literature, the advantages of social media use as well as its risks and abuses are discussed. Social media have been observed as useful as a learning and educational environment, for example to prepare for exams, discuss clinical cases and to share information about internships [11]. As medical students, residents and medical specialists venture into social media, their behaviour herein should also be professional. This adds a new dimension to professional behaviour, sometimes referred to as ‘online professionalism’ [11, 20–22].

Interest in and regulation of online professionalism are increasing in medicine [15, 23–25]. This attention was amplified by the recent publication and subsequent withdrawal of an article in the *Journal of Vascular Surgery*. In this article, the investigators described what they deemed inappropriate social media content generated by surgery fellows and residents [26]. This resulted in the #MedBikini online protest to this study, which was described as having “overtones of sexism and misogyny” [27]. Nevertheless, to the best of our knowledge, hardly any research on online professionalism *from the users’ perspective* has been conducted among medical students, residents and medical specialists in a combined and comparative manner. Consequently, it is unclear how each of the groups perceive online professionalism. This gap in the literature has to be bridged in order determine whether and, if warranted, how online professionalism can potentially be improved in these groups. In this way, targeted interventions could be undertaken to improve social media use in all three groups and potentially have greater impact.

### Aim

This study compares the use of, frequency and nature of what medical students, residents and medical specialists perceive as unprofessional behaviour *on* social media in the Netherlands. Participants’ social media use with regard to their medical training and/or practice and their personal use of social media are included in this study. Four research questions were formulated:

1. Which social media are used and how much time is spent on social media?
2. For what purpose are social media used in medical school and medical practice?
3. What is the perceived frequency of unprofessional behaviour on these social media?
4. What is perceived as inappropriate or unprofessional information on social media?

## Methods

### Data collection

We distributed an adapted and translated survey previously used by Garner and O’Sullivan [23]. We used email recruitment for most potential participants, using Survey Monkey (San Mateo, CA, USA). The invitation to the survey was signed by SG, PB and WvM. Due to university policy, medical students at Maastricht University (UM) were contacted via the intranet, newsletters and social media. Inclusion of the different groups of participants was initiated in December 2015 and closed in March 2017 after we had sent two reminders.

Our survey contained 33 questions: 3 demographic questions and 30 questions regarding social media use. In addition, participants could provide narrative comments on 13 questions, and there was one open-ended question for additional remarks.

### Participants and context

We distributed the survey to medical students in two Dutch medical schools, Leiden University (LU) and UM, with 3844 students in total, 828 residents in three Dutch hospitals and 426 medical specialists at a University Medical Centre. The residents and medical specialists represented a range of individual specialties.

### Data analysis

Descriptive statistics were calculated and a one-way ANOVA with post-hoc adjustment (Tukey) was conducted, using IBM SPSS Statistics for Windows, version 25.0 (IBM Corp., Armonk, NY, USA). Cohen’s *d* was calculated using R (R Core Team, 2019).

Qualitative data analysis was performed by SP, a medical student who is familiar with the use of social media, by extracting the qualitative data from SPSS to Word (Microsoft Corporation, Redmond, WA, USA). Coding was done manually and independently by two researchers (SP and SvL) using open coding [28]. The data was divided into fragments that were compared and grouped into categories with one descriptive code each, which covered the topic so described. Sentences containing information relating to different subjects were coded as separate main or sub-codes.

Next, axial coding by both researchers (SP and SvL) was used to create a list of categories in which some codes were merged in one category and synonyms were removed, to create a code tree of the most important themes. Differences in coding were discussed until consensus was reached.

## Results

Overall, 1354 participants responded: 1102 students, 139 residents and 113 medical specialists. Surveys with 25 or more unanswered questions were excluded from the analysis (note: participants without social media were allowed to skip 15 questions [*n* = 67]). Final analysis included 1262 participants: 1018 medical students (LU: 803, response rate 41.3%; UM: 215, response rate 11.3%), 133 residents and 111 medical specialists. The demographic data are displayed in Tab. 1.

**Table 1:** Overview of data on participants and response rates

	Medical students *n* (%)	Residents *n* (%)	Medical specialists *n* (%)
*Number*	1018	133	111
*Response rate*	28.7%	16.1%	26.1%
*Age in years*			
<20	211 (21.0)	–	–
20–29	781 (77.7)	67 (50.4)	1 (0.9)
30–39	13 (1.3)	65 (48.9)	35 (31.5)
40–49	–	1 (0.8)	34 (30.6)
>50	–	–	41 (36.9)
*Mean age in years (SD)*	22 (2.7)	29 (3.1)	44 (9.0)
*Gender*			
Male	278 (27.3)	50 (37.6)	63 (57.3)
Female	739 (72.7)	83 (62.4)	47 (42.7)
*Year of study*			
1st	155 (15.2)	–	–
2nd	151 (14.8)		
3rd	201 (19.7)		
4th	165 (16.2)		
5th	123 (12.1)		
6th	223 (21.9)		
*Median work years (IQR)*	–	4 (2–6)	10 (4–20)

*SD* standard deviation, *IQR* interquartile range

### Quantitative results

#### Social media usage

**Fig. 1:**
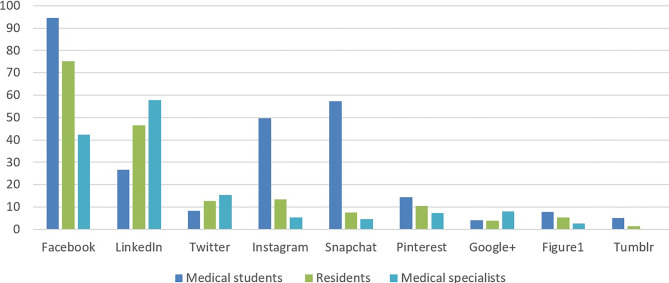
Overview of the use of social media (y‑axis in %)

Fig. 1 provides an overview of social media use among the different groups of participants. The three most frequently used social media differed among the groups. The top three social media reported in the residual ‘other’ group included WhatsApp, YouTube and Reddit. Participation in *medical* social media groups, for example self-created private groups on Facebook, were more common among medical students (90.9%) than among residents (36.2%) and medical specialists (36.4%). The most common use of social media in the medical domain was sharing online material.

Social media usage and age variables were transformed into levels (Tab. 1 and 2). An age difference between the three groups was determined by one-way ANOVA (*F*(2,1073) = 45.11, *p* < 0.001, *ηp*^2^ = 0.08).

**Table 2:** Social media use among the different groups of participants

	Medical students *n* (%)	Residents *n* (%)	Medical specialists *n* (%)
*Uses social media frequently*	973 (95.9)	103 (77.4)	68 (61.3)
*Frequency (hours/week)*			
1: < 1	–	1 (1.0)	2 (3.3)
2: 1–5	315 (34.3)	71 (74.0)	53 (86.9)
3: 6–10	349 (38.0)	17 (17.7)	5 (8.2)
4: 11–15	139 (15.1)	5 (5.2)	1 (1.6)
5: 15–20	49 (5.3)	1 (1.0)	–
6: > 20	67 (7.3)	1 (1.0)	–
*Mean:*	3.13	2.34	2.08
*Standard Deviation:*	1.16	0.74	0.42
*Has encountered a disadvantage of not having social media*	217 (21.5)	20 (15.3)	8 (7.2)
*Uses real name on social media*	954 (95.5)	115 (97.5)	72 (93.5)
*Is aware of the fact that privacy settings change*	754 (75.6)	88 (74.6)	60 (76.9)
*Reads the privacy settings on social media at least once a year and adjusts them if necessary*	259 (26.0)	44 (37.3)	26 (33.8)
*Accepts friend requests from people hardly known*	102 (10.2)	10 (8.5)	8 (10.5)
*Is aware of social media groups in relation to medical training/medical practice*	419 (42.1)	41 (34.7)	22 (28.6)
*Uses social media for:*			
– Sharing online material	545 (56.7)	25 (26.3)	17 (25.4)
– Discussing clinical cases	42 (4.4)	4 (4.2)	3 (4.4)
– Organizing appointments	193 (20.1)	4 (4.3)	6 (8.8)
– Sharing daily schedule	348 (36.3)	8 (8.3)	2 (2.9)

The definition of the aspect ‘frequently’ using social media was determined by the participants themselves (Tab. 2). A Tukey post hoc test revealed that the time spent on social media among medical students (6–10 h/week) was higher than among residents (1–5 h/week, *p* < 0.001, Cohen’s *d* = −0.70) or medical specialists (1–5 h/week, *p* < 0.001, Cohen’s *d* = −0.93). There was no difference between residents and medical specialists (*p* = 0.314). Comparing the time spent on social media between the age groups showed a difference (*F*(4,1064) = 22.38, *p* < 0.001, *ηp*^2^ = 0.08). Time spent on social media among participants younger than 20 years (*p* < 0.001) and between 20 and 29 years (*p* < 0.005) differed from all other age groups. There was no difference in time spent on social media among the age groups 30 years of age and older.

#### Frequency of perceived unprofessional behaviour on social media

Approximately one-third of the medical students (31.3%) self-reported the presence of information *about themselves* on social media that they perceived as unprofessional from a professional perspective. This percentage was lower among residents (19.7%) and medical specialists (4.1%). When asked about unprofessional behaviour on social media among *their colleagues*, these percentages were higher: medical students (48.5%), residents (57.3%) and medical specialist (32.5%). Postings of their own unprofessional behaviour on social media that they perceived as potentially harmful for their future careers were reported by 4.9% of medical students, 3.4% of residents and none of the medical specialists.

#### Social media guidelines

Respondents were asked to what extent they agreed with a series of statements relating to social media, fitness to practice and national guidelines, as depicted in the Electronic Supplementary Material (ESM), Appendix 1. A majority of respondents agreed that behaviour outside the clinical environment, including social media, could impact fitness to practice (medical students (62.9%), residents (64.8%) and medical specialists (79.3%)). Most medical students (71.7%) stated that they understand what is classified as unacceptable behaviour. This is to a lesser extent the case among residents (57.0%) and medical specialists (58.6%). The awareness of the existence of guidelines on professional behaviour among the participants showed a significant difference (*F*(2,1218) = 6.78, *p* = 0.001, *ηp*^2^ = 0.019). Awareness is highest among medical specialists (43.6%) as compared to medical students (24.5%,* p* = 0.001, Cohen’s *d* = 0.35) and residents (27.1%,* p* = 0.006, Cohen’s *d* = 0.38).

#### Addressing colleagues

The results indicated that it is more likely that a colleague will be addressed when sharing *patient information* than when sharing inappropriate *personal information* on social media. Medical specialists reported to be more likely to address colleagues regarding observed unprofessional social media behaviour than were residents or students. When inappropriate information about patients is shared on social media, the responses among all groups were similar, with over 80% agreeing to address this issue with their colleagues.

### Qualitative results

Independent coding of narrative comments resulted in an 85% interrater agreement. The remaining 15% of comments were (re)coded after face-to-face sessions until consensus was reached between SP and SvL. Of the comments, 7.3% could not be categorized, because the text did not contain words, was a question itself or referred to other answers. Illustrative participants’ quotes are reproduced in ESM, Appendix 2.

#### Purpose of social media use

For all three groups social media were mainly used to gather information that would otherwise be missed, such as study-related information, vacancies and events, and as a way to keep in contact with others. Students joined *local* social media groups (e.g., medical faculty/medical student association groups) eight times more frequently than *(inter)national* medical groups. The residents and medical specialists also joined *local *social media groups, for example their old medical faculty groups and current hospital groups, more often than *(inter)national* medical groups.

#### Information perceived as unprofessional

Narrative comments on unprofessional information about *themselves *on social media were provided by 51 medical students, 4 residents and none of the medical specialists. This includes, for example, pictures of themselves drunk. Narrative comments on unprofessional information about *colleagues* on social media were provided by 360 medical students, 44 residents and 18 medical specialists. Whereas the topics identified were similar for *themselves *and *colleagues, *the order based on frequency of reporting differed among the groups (see Tab. S1 in ESM).

Alcohol abuse posts were the most frequently mentioned among students (*n* = 287), followed by (costume) party posts (*n* = 116). Clinical work-related posts among students were often mentioned in the context of the clinical phase of medical school or anatomy courses. Among residents and medical specialists, clinical information in which patients and/or colleagues were mentioned was perceived as unprofessional, since this could breach professional confidentiality. Among residents there was little difference between the top two subjects: alcohol abuse posts were mentioned 23 times and sexually suggestive posts 19 times. Medical specialists also frequently mentioned political posts as unprofessional. Coded ‘other’ items included a wide range of subjects such as inappropriate gestures and remarks, smoking and drug use.

#### Acceptability of information

The survey contained four questions—with statements about personal life, personal work experiences, colleagues and patients—exploring the acceptability of social media posts. Among the three groups, we found agreement concerning reasons for professional acceptability of information on social media. The common prerequisite for sharing information was that this be done by sending a *private message* rather than posting it in the *public domain*. In the posted subject or case, anonymity must be guaranteed.

#### Addressing colleagues

We found that the willingness of all participant groups to address what they perceived as unprofessional behaviour in others depended on the hierarchy and the nature of their relations with others. However, the majority of the medical specialists stated that they would not address others regarding what they perceived as unprofessional social media posts because they did not see it as their responsibility or because they feared it would harm existing interpersonal relationships.

## Discussion

This study provides unique insights into so-called ‘online professionalism’ in the context of medical education and clinical performance, from the self-reported perspective of three groups of medical healthcare professionals. The majority of the medical student and residents’ respondents in our study was female, and representative of the contemporary female-to-male ratio among future physicians in our country.

Social media use was found to be highest among students, potentially explained by differences in social media use based on age, as previously suggested [29]. The most popular social media differed between students and residents (Facebook), on the one hand, and medical specialists (LinkedIn) on the other. This aligns with an Australian study reporting that 99.4% of medical student participants reported Facebook use [24].

The majority of respondents in all groups were unaware of the national guidelines for professional behaviour. Information or pictures perceived to be unprofessional by the respondents themselves included those related to alcohol abuse, partying and sexually suggestive posts, a finding confirmed by several other studies [25, 30]. Perhaps some of their opinions have now changed due the debate initiated by the #MedBikini movement, which occurred after our data collection. Social media posts were perceived as acceptable from the participants’ perspective if the message was private and could only be read by the person to whom it was directed. However, one can ask whether this one-on-one communication can still be considered as social media use, despite the use of a social media tool.

Participants reported being most likely to call out colleagues as unprofessional in response to posting of patient data, but this depended on the nature of the interpersonal relationship with the colleagues involved. Medical students admitted they are more likely to address their peers and not their supervisors. A study among medical students underscores this and showed that not knowing how to respond to lapses, believing addressing them is futile or fearing retaliation, prevented students from addressing peers and staff [31]. Potentially this also applies to residents who are supervised by medical specialists. This creates the risk that those healthcare professionals who post unprofessional content unintentionally, *persist* in doing so, since no feedback is provided, subsequently continuing to pose a risk to patient safety and the hospitals’ reputation.

### Strengths and limitations

A unique aspect of this study is that it compared the use and *self-reported *perceptions of social media among medical students, residents and medical specialists, and not the authors’ interpretation. Furthermore, the female-to-male ratio among respondents was representative of that in contemporary Dutch medical schools and postgraduate training programmes. However, the mean age of medical specialists, 44 years, was relatively young, and more males than females participated. It is possible that older specialists did not participate in this study and their opinions of and use of social media were underrepresented in the results. The participants often mentioned WhatsApp as a social medium, which was not included as a social medium in the survey. Although minimization of selection bias was strived for by contacting most participants via email, medical students at UM were partly recruited through social media, introducing some selection bias, and at least partly explaining the lower response.

### Further research

The *exact *nature of issues related to social media that medical professionals consider more disturbing than others, and the reasons for these perceptions are yet to be unravelled. Furthermore, it is unknown how medical under- and postgraduate education and training can play a role in increasing the readiness to address colleagues regarding unprofessional (online) social media behaviour and awareness of related guidelines. It might be valuable to reassess these perceptions over time to see whether these perceptions have changed. The frequency and consequences of unprofessional online behaviour of other healthcare professionals, including nurses, has also not been studied here.

## Conclusion

Social media use among medical students, residents and medical specialists is common. The majority of participants use social media to gather and share information that otherwise would be missed and to keep in contact with others. Among the three groups, medical students spent the most time on social media. Participants acknowledged that inappropriate information was commonly posted on social media. But they were more likely to perceive it as inappropriate when posted by peers and colleagues than by themselves. Addressing colleagues about unprofessional online behaviour is most likely to occur when patient privacy and confidentiality is breached. However, the respondents mentioned that this is difficult in practice. Medical educators should thus equip trainees with the skills to provide feedback to address such issues in a constructive manner by using already existing (online) guidelines on professional behaviour in the undergraduate and postgraduate medical curriculum.

## Supplementary Information

**Appendix 1** Statements on social media use and awareness of guidelines: extent of agreement

**Appendix 2** Participants? quotes concerning social media use

**Appendix 3** Table S1 Top five topics of social media posts perceived as unprofessional
