# Supplementary material for: Perception of social media behaviour among medical students, residents and medical specialists
Source: Perspect Med Educ. 2021 Apr 7;10(4):215–21. doi: 10.1007/s40037-021-00660-1 (PMC8368941; doi:10.1007/s40037-021-00660-1)
Supplement: Supplementary file 1 — Appendix 1 Statements on social media use and awareness of guidelines: extent of agreement [file 40037_2021_660_MOESM1_ESM.docx]

**Electronic Supplementary Material**

**Appendix 1:** Statements on social media use and awareness of guidelines: extent of agreement

|  | **Medical students**  ***n* (%)** | | | **Residents**  ***n* (%)** | | | **Medical specialists**  ***n* (%)** | | |
| --- | --- | --- | --- | --- | --- | --- | --- | --- | --- |
| **Answer** | Strongly agree | Neither agree, nor disagree | Strongly disagree | Strongly agree | Neither agree, nor disagree | Strongly disagree | Strongly agree | Neither agree, nor disagree | Strongly disagree |
| **I use my social media account(s) only for private purposes** | 524 (54.4) | 297 (30.8) | 143 (14.8) | 74 (65.5) | 22 (19.5) | 17 (15.0) | 25 (31.6) | 27 (34.2) | 27 (34.2) |
| **I never talk about my friends in my messages on social media** | 162 (16.8) | 286 (29.7) | 515 (53.5) | 26 (23.0) | 35 (31.0) | 52 (46.0) | 44 (55.7) | 21 (26.6) | 14 (17.7) |
| **What happens on websites such as Facebook is separate from what happens in medical school** | 246 (25.1) | 401 (40.8) | 335 (34.1) | 26 (20.2) | 46 (35.7) | 57 (44.2) | 13 (11.7) | 23 (20.7) | 75 (67.6) |
| **Behaviour**  **outside the clinical environment, including social media, could have an impact on fitness to practice** | 617 (62.9) | 257 (26.2) | 107 (10.9) | 83 (64.8) | 36 (28.1) | 9  (7.0) | 88 (79.3) | 16 (14.4) | 7  (6.3) |
| **I understand what the medical school / a medical specialist would classify as unacceptable behaviour** | 697 (71.1) | 193 (19.7) | 90  (9.2) | 73 (57.0) | 39 (30.5) | 16 (12.5) | 65 (58.6) | 34 (30.6) | 12 (10.8) |
| **I am aware of the national guidelines on personal and professional behaviour** | 241 (24.5) | 268 (27.3) | 473  (48.2) | 35 (27.1) | 24 (18.6) | 70 (54.3) | 48 (43.6) | 21 (19.1) | 41 (37.3) |
