# Supplementary material for: Perception of social media behaviour among medical students, residents and medical specialists
Source: Perspect Med Educ. 2021 Apr 7;10(4):215–21. doi: 10.1007/s40037-021-00660-1 (PMC8368941; doi:10.1007/s40037-021-00660-1)
Supplement: Supplementary file 2 — Appendix 2 Participants? quotes concerning social media use [file 40037_2021_660_MOESM2_ESM.docx]

**Electronic Supplementary MaterialAppendix 2:** Participants’ quotes concerning social media use

| **About** |  | **Participants’ quotes** |
| --- | --- | --- |
| **Purpose of social media use** | | |
|  | Medical_student_0670 | When I started studying medicine, it was a disadvantage for me to not have a Facebook account. Through Facebook, important, study-related information was shared, for example by the study association in a specific, dedicated Facebook group. I then created a Facebook account in order not to miss any important information |
|  | Resident_121 | Before I had a LinkedIn account, I noticed that I was less findable for the employers where I came to apply |
| **Information perceived as unprofessional** | | |
|  | Medical_student_0203 | I have shared videos or articles on my Facebook page that have controversial opinions about social issues such as the refugee crisis, racism, and feminism |
|  | Medical_student_0985 | For example, many pictures of people who are very drunk, with a lot of drinks in the picture, with weird texts in the photo etc. Or for example photos that are very exposed |
|  | Resident_001 | Photos with patients in the operating room (usually not recognizable), photos with patients in developing countries (often recognizable), comments about the average intelligence of patients from a certain region around the workplace, that someone had caught a well-known athlete at the emergency room at put a link to the news item on social media |
| **Acceptability of information on social media** | | |
|  | Medical_student_0049 | Never put the negative messages on social media. Also, because it concerns patients in this case, and I would not put anything on social media at all, even if it is a positive message. This is also very close to harming your professional confidentiality |
|  | Medical_student_0256 | You have to be careful with this, I think positive statements are accepted, except when it concerns the appearance of someone. I would be very careful with negative messages, first and foremost because it could be better face-to-face, so that no other people can watch / listen in. To be sure that it will not end up with that person |
|  | Resident_005 | If it is a private message and the patient is not mentioned by name, I think it is acceptable that expressions of appreciation (a pie, a hug) are shared. The same applies to brief reporting of an interesting case (I recently diagnosed a sarcoidosis for the first time!). A case discussion with all the details I think goes too far, but there is no hard line |
| **Addressing colleagues about unprofessional posts** | | |
|  | Medical_student_0631 | As a medical student during my clerkships I would only do this for students / friends I know well, against unknowns or people you depend on (such as residents or medical specialists) I would not address this |
|  | Resident_084 | When inappropriate information about patients is shared, I will address a colleague more quickly. However, this is generally very dependent on which colleague this is and how my relationship with him/her is |
|  | Medical_specialist_031 | Depends completely, I am not a headmaster, and what is permissible and prevalent in this area is rapidly changing |
