# Supplementary material for: Perception of social media behaviour among medical students, residents and medical specialists
Source: Perspect Med Educ. 2021 Apr 7;10(4):215–21. doi: 10.1007/s40037-021-00660-1 (PMC8368941; doi:10.1007/s40037-021-00660-1)
Supplement: Supplementary file 3 — Appendix 3 Table S1 Top five topics of social media posts perceived as unprofessional [file 40037_2021_660_MOESM3_ESM.docx]

**Electronic Supplementary Material**

**Table S1:** Top five topics of social media posts perceived as unprofessional

| **Information perceived as unprofessional on social media** | | |
| --- | --- | --- |
| (*n* = 411) | (*n*= 48) | (*n*= 18) |
| **Medical students** | **Residents** | **Medical specialists** |
| 1. Alcohol abuse 2. (Costume) Party 3. Sexually suggestive 4. Other 5. Clinical work | 1. Alcohol abuse 2. Sexually suggestive 3. Clinical work 4. Other 5. (Costume) Party | 1. (Costume) Party 2. Clinical work 3. Alcohol abuse 4. Sexually suggestive 5. Politics/Opinions |
